# Accelerated Polyethylene Terephthalate (PET) Enzymatic Degradation by Room Temperature Alkali Pre‐treatment for Reduced Polymer Crystallinity

**DOI:** 10.1002/cbic.202200503

**Published:** 2022-11-30

**Authors:** Sariah Giraldo‐Narcizo, Nihal Guenani, Ana María Sánchez‐Pérez, Antonio Guerrero

**Affiliations:** ^1^ Institute of Advanced Materials (INAM) Universitat Jaume I 12006 Castelló Spain; ^2^ Faculty of Health Sciences Universitat Jaume I 12006 Castelló Spain

**Keywords:** biocatalysis, enzymes, enzyme catalysis, polymers

## Abstract

Polyethylene terephthalate (PET) is the most widely employed plastic for single‐use applications. The use of enzymes isolated from microorganisms, such as PETase with the capacity to hydrolyze PET into its monomers, represents a promising method for its sustainable recycling. However, the accessibility of the enzyme to the hydrolysable bonds is an important challenge that needs to be addressed for effective biodegradation of postconsumer PET. Here, we combined an alkali pre‐treatment (25 °C) with PETase incubation (30 °C) with post‐consumed PET bottles. The pre‐treatment modifies the surface of the plastic and decreases its crystallinity enabling the access of the enzyme to the hydrolysable chemical bonds. When the alkali pre‐treatment is incorporated into the enzymatic process the degradation yields increase more than one order of magnitude reaching values comparable to those obtained during heating/cooling cycles. Our results show energetic advantages over other reported pre‐treatments and open new avenues for sustainable PET recycling.

## Graphical abstract

**Alkali pre‐treatment** at mild temperatures modifies the surface of polyethylene terephthalate (PET) samples and reduces the crystallinity. These two effects improve the access of PETase to hydrolysable chemical bonds increasing the production of terephthalic acid (TPA) and monohydroxyethyl terephthalate (MHET) by one order of magnitude.

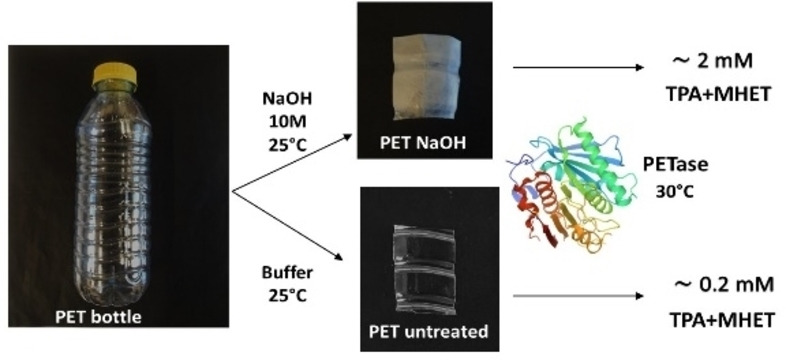

## Introduction

Plastics are very useful materials, however, the gigantic worldwide production and their uncontrolled disposal have led to unmeasurable amounts of solid waste and pollution in rivers and seas with great impacts in ecosystems.^[1]^ The solution to this problem needs to be addressed from several facets including the production of bio‐degradable polymers with adequate physical properties, improving recycling processes of existing polymers, modification of environmental policies, and promotion of self‐awareness of consumers to reduce, reuse and recycle the plastic products.

In 2019 global plastic production reached more than 368 million tons globally,^[2]^ and any proposed recycling strategy needs to be scalable and must take into account the energy required in the process.^[2]^ Traditional methods such as mechanical, chemical, pyrolysis, solvolysis recycling are costly, contaminating or are limited technically in separation of blends and multilayers.[^3^, ^4^] Enzymatic degradation is emerging as a potential alternative route for monomer recovery, however the accessibility to the hydrolysable bond makes it hardly applicable to effective recycling. Therefore, is has been suggested that only the combination of different technologies can address the plastic waste recycling problem.^[3]^

PET is considered the archetypical polymer that has been recycled successfully and a model system for other polymer families. Despite the high recycling rate of PET, this is far from circular economy; mechanical recycling leads to inferior physical properties in comparison to the virgin polymer, thus it is only viable for a limited number of cycles.^[3]^

Different chemical processes can be used for PET degradation including glycolysis, methanolysis and hydrolysis.^[5]^ The latter is the only process that yields terephthalic acid (TPA) and ethylene glycol (EG), the monomers to generate new PET polymers. However, all these processes involve high temperatures and/or high pressures.^[6]^ These conditions require high energy consumption to increase the reactors temperature over 100 °C. For instance, PET thermolysis generates useful double bond‐terminated fragments but need temperatures in the range of 250–300 °C.^[7]^ These high energy requirements hamper their application as a sustainable recycling method of postconsumer PET (for a review in the subject see ref. [8]).

Interestingly, in the last years the exciting discovery of microorganisms with capacity to degrade PET into monomers operating under very mild conditions have opened new research avenues.[^9^, ^10^, ^11^] The rate of polymer biodegradation depends on several factors such as polymer molecular weights, and degree of crystallinity.[^12^, ^13^] Indeed, the accessibility of the enzyme to the polymer bonds to cleave is one of the major challenges in highly crystalline polymers that forms highly packed domains. Thus, active enzymes for PET depolymerization show that their catalytic activities are very low hindering their application in commercial biodegradation. The activity of the enzymes has been improved by introducing different mutations but degradation of highly crystalline post‐consumer PET remains challenging.[^14^, ^15^, ^16^] Therefore, additional chemical or biological processes are required to increase their activity.

Different pre‐treatment processes have been employed to increase the enzymatic activity such as heating prior to enzymatic degradation that reduces PET crystallinity, see below in the discussion, or use of other proteins, like hydrophobins to improve PETase accessibility in high crystallinity PET.^[17]^ In spite of these advances, pretreatments to make biodegradation systems efficient still suffer from high energy requirements or involve complex treatments.

In this work, we aim to explore sustainable processes to biodegrade postconsumer PET into monomers that could be repolymerized into virgin polymer for effective circular economy. We propose the use of an alkali pre‐treatment at very mild temperatures to improve the access of PETase onto the polymer surface. We found that under these conditions the yield of depolymerized monomers production is increased by a factor of 10.

## Results and Discussion

**Figure 1:**
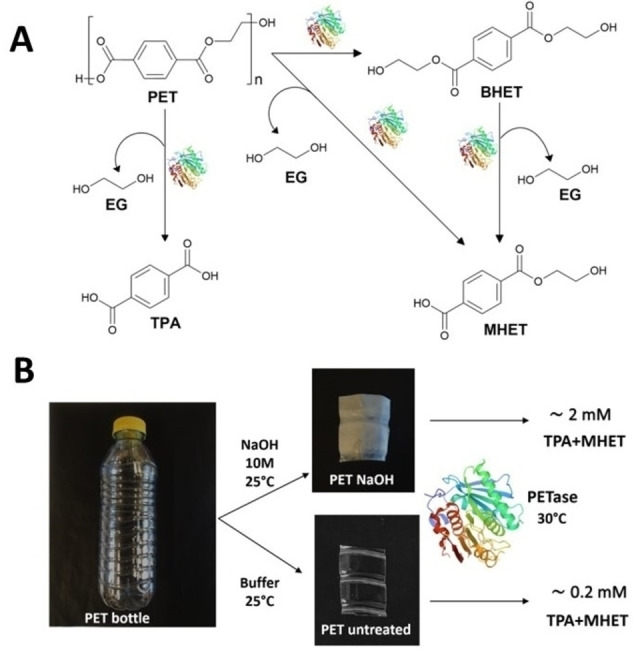
(A) Scheme of the different PET hydrolysis pathways catalyzed by PETase. (B) Schematic diagram of experimental design comparing PETase catalytic efficiency with or without alkaline pre‐treatment of the PET substrate.

We base our study on the well‐known PETase from *Ideonella sakaiensis* enzymatic activity.[^18^, ^19^] PETase can follow different pathways to produce TPA and monohydroxyethyl terephthalate (MHET) and ethylene Glycol (EG)[^19^, ^20^, ^21^] (Figure 1A). Here, we compare the activity of this enzyme on untreated highly crystalline (∼35 %) post‐consumer PET water bottle with its activity on a similar substrate that has been previously treated with an alkali (NaOH, 10 M) at room temperature (25 °C) during 24 h. The PETase incubation is also carried out on both substrates at mild temperatures (30 °C), the optimal temperature for PETase.[^22^, ^23^]

First, we observed visual differences between untreated and NaOH pre‐treated samples (Figure 1B). Whilst the untreated sample is transparent and colorless the NaOH pre‐treatment on the PET sample leads to an opaque substrate and white in color.

**Figure 2:**
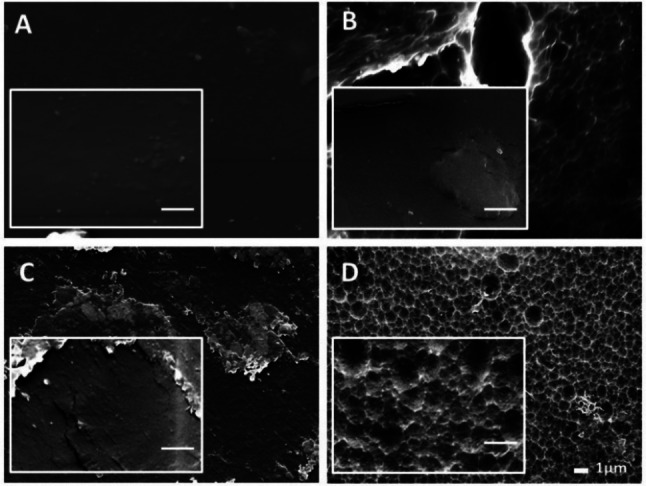
SEM measures of post‐consumer PET water bottle surface. (A) PET bottle untreated. (B) PET bottle pretreated with 10 M NaOH. (C) Untreated PET bottle incubated with PETase. (D) PET bottle pretreated with alkali followed by PETase incubation. Scale bar: A‐D and insets 1 μm.

We next examine the surface of the PET plastic samples by Scanning Electronic Microscopy (SEM) of plastic specimens before and after the enzymatic treatment. We find that the post‐consumer PET plastic that has not received a pre‐treatment shows a very smooth surface with the presence of small crystalline granules embedded into the polymer matrix (Figure 2A). However, the sample that has been treated with NaOH shows a rough surface with texture in the range of tens of microns (Figure 2B).

The two plastic substrates were incubated with the enzyme during 96 h at 30 °C and were also analyzed by SEM. The morphology of the non‐pre‐treated PET that has been incubated with the PETase (Figure 2C) shows that the surface is also affected with formation of scales indicating that the enzyme has worked through the surface of the polymer. Alternatively, the combination of the alkali pretreatment with enzymatic degradation (Figure 2D) leads to a much severe surface degradation as compared with the rest of the samples. These findings suggest that the pretreatment seem to prepare the surface for improved enzyme accessibility.

**Figure 3:**
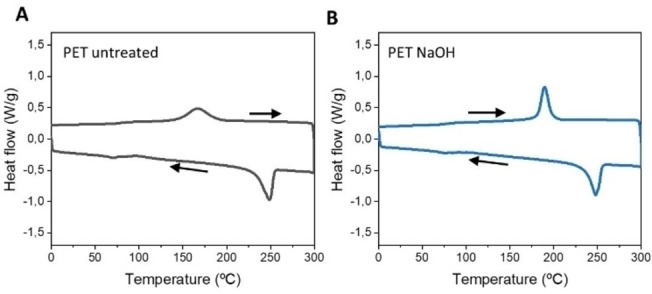
Differential scanning calorimetry (DSC) for crystallinity evaluation of (A) Untreated PET bottle and (B) PET bottle pre‐treated with NaOH. Arrows indicate the variation of the temperature to show the heating or cooling curves.

Given the morphological alterations we next set to evaluate possible changes in the crystallinity of the plastic pieces. To that end, we measured the Differential Scanning Calorimetry (DSC) of the untreated and NaOH‐treated PET samples (Figure 3). Using DSC the crystallinity is calculated by comparison of the heat required to melt the crystalline fractions, melting enthalpy corresponds to the peak with positive values, with the heat that the sample takes from the system during crystallization during cooling, cold crystallization enthalpy corresponds to peak with negatives values.^[23]^ We observed that PET crystallinity was reduced from 33.70±0.05 % to 27.68±0.34 % after the NaOH treatment. Although this reduction may not seem high, reported results suggest that even a small reduction in the crystallinity of the polymer substrate is sufficient to increase the catalytic activity of the enzymes.^[24]^

**Figure 4:**
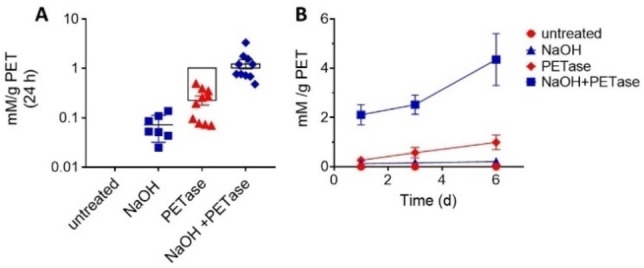
HPLC measurements of TPA+MHET products. A) TPA+MHET (mM) in different conditions estimated to 24 h. B) TPA+MHET (mM) accumulation up to 6‐days incubation period. Data are estimated per gram of PET, using 2 mg enzyme/g PET and expressed as mean±SEM. Calculated from a standard curve (Figure S1).

To evaluate if the surface morphological alterations leads to improved hydrolysis during enzymatic treatment, we quantified the MHET+TPA production in each sample by using High Performance Liquid Chromatography (HPLC). Separation methods are difficult due to the low solubility of the products and the presence of oligomers that block the HPLC columns. For this reason, we use a reported method in which the peak for BHET is well resolved and MHET and TPA peaks show at the same retention time (Figure S1).^[25]^ The HPLC results (Figure 4A) of a reference sample of untreated post‐consumer PET sample with no enzymatic treatment confirms that PET is not soluble in water and methanol and no products were detected. Alternatively, we found that the NaOH treatment alone with no enzymatic treatment only produces low quantities of MHET+TPA **0.12**±**0.01 mM/g PET**, note that a semi‐log graph is represented to enable a better comparison between the 4 samples. Under these mild conditions the NaOH treatment releases PET oligomers, with a measured weight loss of about 5 %, but no elementary products such as TPA and EG are detected by Nuclear Magnetic Resonance, a technique able to identify oligomers but less sensitive than HPLC. At this point it is important to note that PET leads to complete hydrolysis using a catalyst transfer and increasing the temperature to 90 °C.^[26]^ All these results confirm that the kinetics of the hydrolysis reaction using our alkali treatment at room temperature are very low. Then, there is a need for the additional enzymatic treatment to accelerate the degradation of consumer PET.

Enzymatic treatment of post‐consumer PET with the PETase was used directly on untreated PET. We obtained results within reported values with mean MHET+TPA concentration of **0.25±0.11 mM/g PET** during the first 24 h. This result agrees with a production of MHET+TPA that depends on the crystallinity of the substrate, see Table 1. Indeed, our results are lower than those obtained using amorphous PET film with crystallinity of 2–3 %^[27]^ but about 10‐fold higher than those obtained by PETase incubation with higher crystallinity bottles (∼45 %).[^23^, ^28^, ^29^] Here, it is important to mention that amorphous PET is not relevant for consumer applications but the trend illustrates the potential role of enzymatic degradation if the enzymatic accessibility issue is circumvented. Therefore, we highlight that PET crystallinity is a great determinant in the efficacy of the enzymatic methods to degrade the polymer. In our case, the untreated commercial PET bottle sample with crystallinity of 33 % shows intermediate production of MHET+TPA.

**Table 1:** Comparative results of PETase activity after 24 h incubation time with regard to the crystallinity fractions (amorphous vs high crystalline PET) and the effect of pretreatments.

Entry	Substrate	PET crystallinity	Pretreatment	Reaction temp.	Volume [μL]	PET loading [mg]	Enzyme loading [μM]	Enzyme/PET	Incubation conditions: buffer	MHET+TPA concentration	Yield of TPA+MHET [%]	Ref.
1	Amorphous PET film (Goodfellow)	2‐3 %	No	30 °C	not reported	not reported	not reported	2 mg enzyme/g PET	20 mM Tris HCl, 100 mM NaCl (pH 7.5)	0.33 mM	–	[25]
2	Post‐consumer Coca‐Cola PET bottle 6 mm diameter pieces	45 %	No	30 °C	300	∼25^[b]^	0.05	0.2 mg enzyme/g PET	glycine‐NaOH buffer (50 mM, pH 9.0)	1.2 μM	0.3×10^−3^	[29]
3	Post‐consumer Coca‐Cola PET bottle 6 mm diameter pieces	45 %	No	30 °C	300	∼25^[b]^	0.20	8×10^−2^ mg enzyme/g PET	glycine‐NaOH buffer (50 mM, pH 9.0).	5.5 μM	1.3×10^−3^	[28]
4	PET film, 6 mm diameter pieces (UBIGEO)	42 %	No	30 °C	300	∼25^[b]^	0.50	2×10^−2^ mg enzyme/g PET	glycine/NaOH buffer (50 mM pH 9.0)	6.6 μM	1.5×10^−3^	[23]
5	Post‐consumer PET bottle	33 %	No	30 °C	200	∼80	∼25	2 mg enzyme/g PET	glycine/NaOH buffer (50 mM, pH 9.0)	0.25 mM	1×10^−2^	Our study
6	Post‐consumer PET bottle	27 %	NaOH 10 M (24 h)	30 °C	200	∼80	∼25	2 mg enzyme/g PET	glycine‐NaOH buffer (50 mM, pH 9.0)	2.1 mM	0.1	Our study
7	Post‐consumer PET bottle 6 mm diameter pieces	30 % (Initial) 2 % (treated)	Heat (20 min, 290 °C)	50 °C	600	25	0.20	0.15 mg enzyme/g PET	100 mM KH_2_PO_4_‐ NaOH buffer (pH 8.0)	0.5 mM	0.2	[30]
8	PET powder (Goodfellow)	38 %	No	30 °C	500	15	1.81	2 mg enzyme/g PET	300 mM NaCl, 20 mM Tris HCl, pH 8.0	2.7 mM	1.8	[22]
9	PET powder	35 % (Initial) Powder (treated)	Ball milling (5 min)	55 °C	450	300	∼130	6.5 mg enzyme/g PET	0.1 M sodium phosphate buffer (pH 7.3)	324 mM	9.25^[c]^	[31]
10	PET fibers	38.8 %	Hydrophobins^[a]^ (3 h)	30 °C	200	3	0.60	1.3 mg enzyme/g PET	50 mM phosphate buffer (pH 8.0)	14 mM	17.7	[32]

[a] Surface‐active proteins enhanced PETase hydrolysis of semi‐crystalline PET fiber and high‐crystalline PET bottle. [b] We estimate that 6 mm diameter pieces weight 25 mg. [c] Enzyme used was a commercial HiC.

As expected from the surface analysis and crystallinity results, we obtained best yields of MHET+TPA when the alkali pre‐treatment is combined with the PETase incubation, the mean products concentration of **2.11**±**0.41 mM/g PET** is obtained during the first 24 h. Overall, the NaOH pre‐treatment increases the enzymatic activity by one order of magnitude as compared to the sample that was untreated with NaOH. We attribute this effect to the better access of the enzyme to the hydrolysable bonds due to a reduction in the crystallinity of the PET sample.

In order to evaluate the stability of the enzymes we completed incubations over long periods of time (Figure 4B). We observed that the production of TPA+MHET increased over the 6 days incubation period. However, the accumulated products (TPA+MHET) slows down during the following days. The results are in good agreement with other studies with incubation periods that were completed over 7^[22]^ and 10^[23]^ days. Our results suggest that the enzyme is still active up to 6 days, but it loses activity after 24 h.

To discuss the effectiveness of different strategies reported to improve PET biodegradation, we show in Table 1 some representative examples with detailed experimental conditions, together with the reported/calculated yields of products (TPA+MHET) after 24 h incubation time at 30 °C. The limitation of this general comparison is that, often, the yield is not reported, or the publication lack the essential information to calculate it, i. e. reaction volume or PET loading.

Plastics with high crystallinity show very low yields, that PETase modifications or mutations do not improve significantly (Entries 2–5). For example, the yield PET degradation was increased 11 fold (a maximum yield of ∼6×10^−3^ %) by the addition of alternating glutamic and lysine residues to the PETase.^[28]^ Also, addition of monomers onto the PETase could increase 1.5‐fold its activity to a maximum of 0.7×10^−3^ % yield.^[29]^ Moreover, rational design of mutant PETase resulted in more thermostable enzymes, the best of which gave a yield of 17.9×10^−3^ % at 40 °C by PETaseS121S, D186H, R280 A mutant. This data indicates that, up to date, no enzyme modification or mutation has obtained significant yields on postconsumer PET, clearly supporting that pre‐treatment is necessary.

In our study, alkali pre‐treatment (Entry 6) increases degradation to a yield of TPA+MHET in the range of 1×10^−2^ % after 24 h. Although this yield is still very low, alkali pre‐treatment is not a high energy demanding process. Alternatively, PET crystallinity can be reduced to 2 % (Entry 7) by two heating/cooling cycles at 290 °C,^[30]^ this method involves large energy consumption, making difficult to scale the process up for tons of plastic waste. Other strategies chose to increase the surface accessibility to enzymes. For instance, high crystalline powder (Entry 8)^[22]^ or post‐consumer PET treated by ball milling (Entry 9)^[31]^ give a yield of approximately 2 % after 24 h incubation with wild type PETase. Ball milling reduces postconsumer plastic to a micron size particle powder, and should not be mistaken with a general grinding process widely used in the plastic recycling industry that reduces plastic waste to centimeters particles.

Ball milling is also a highly energy demanding treatment which may not be easily applicable at large scale. In fact, the reduction in particle size takes place by the large frictional forces that occur internally during the process. Finally, novel strategy to increase accessibility involves PET reduction to fibers (Entry 10), followed by hydrophobins RolA (*Aspergillus oryzae*) and HGFI (*Grifola frondose*) enzymes incubation prior to PETase.^[32]^ The pre‐treatment with these enzymes notably modify the surface of PET fibers, increasing the amorphous regions for hydrolytic enzyme attack. In these conditions, up to 17.2 % yield is reported (**14 mM** TPA+MHET) in the first 24 h. The method also relies on fibers of PET which would require additional processes to produce them.

## Conclusion

Our data supports that the proposed mild temperature alkali pre‐treatment modifies the surface of the substrate and reduces the crystallinity of the PET substrates. Overall, the treatment enables an efficient access of the PETase to the reactive chemical bonds allowing an efficient chemical degradation of the PET with formation of MHET and TPA. The method provides reaction yields similar to other treatments more energy intensive (heating/cooling cycles), therefore offering advantages regarding its lower energy requirement and affordable chemicals (NaOH). As highlighted in this work none of the reported degradation conditions (including our conditions) are yet applicable to large scale of plastic waste treatment. Reported degradation yields are low after 24 h of reaction when untreated high crystalline post‐consumer plastic is used. This study aligns with previous reports, supporting that effective plastic recycling must include the combination of several methodologies, chemical, physical and biological approaches. Further optimization studies to combine large plastic surface area and alkali pretreatment are warranted to design the protocol that best suits PET waste effective management.

## Experimental Section

**Enzyme expression and purification**: OverExpressTM *E. coli* C41 (DE3) (Lucigen) cells were transformed with pET21a(+) plasmid constructed with PETase(GenScript Biotech). Single colonies were inoculated into a starter culture of Luria Broth (LB) media containing 100 μg/mL ampicillin and grown at 37 °C, 250 rpm overnight. Following day, the culture was inoculated at a 100‐fold dilution into a 2xYT medium containing 100 μg/mL ampicillin and grown at 37 °C until the optical density measured at 600 nM (OD600) reached 0.6–0.8. Protein expression was induced by addition of isopropyl β‐D‐1‐thiogalactopyranoside (IPTG) to a final concentration of 0.5 mM. Bacteria were maintained at 20 °C, 200 rpm for 18 hours following IPTG induction, harvested by centrifugation ((5000 x g, 5 min, 4 °C), and stored at −20 °C until purification. For protein isolation, bacterial pellets were resuspended in 2 mL of lysis buffer (20 mM sodium phosphate, 0.5 M NaCl, 10 mM Imidazole, pH 7.4) and lysed by sonication (15 min with cycles of 10 s). Lysate was clarified by centrifugation at 20000×*g* for 45 minutes at 4 °C and applied to an immobilized metal ion affinity column (IMAC) His SpinTrap columns (GE Healthcare). Protein was eluted with phosphate buffer, 500 mM Imidazole, pH 7.4. The resulting fractions containing proteins of interest were applied to a PD MiniTrap G‐25 size exclusion (SEC) column (GE Healthcare), equilibrated with 50 mM Glycine/NaOH pH 9.0. The concentration and purity of the obtained proteins was assayed by Nanodrop Spectrophotometer and sodium dodecyl sulfate‐polyacrylamide gel electrophoresis (SDS‐PAGE), followed by silver staining or transfer to membrane for Western blot using primary antibody against the hexa‐histidine epitope tag (Invitrogen).

**NaOH pretreatment of PET‐bottle**: Plastic pieces of about 1×1 cm^2^ are cut from a postconsumer PET bottle in the area 3–10 cm above the base of the bottle (see Figure 1B). Untreated samples are washed with distilled water and subsequently dried in a vacuum oven for 20 h at 40 °C and weighed. Samples subjected to alkali treatment are immersed into NaOH (10 M) during 24 h at room temperature, washed with distilled water, vacuum dried and weighed.

**Enzymatic degradation of PET**: Untreated and NaOH pretreated pieces were immersed in 200 μL glycine‐NaOH buffer (50 mM, pH 9.0) with purified enzymes (2 mg enzyme/g PET) at 30 °C for 1–6 d. After centrifuging and filtering, the supernatant was applied to HPLC for the determination of PET decomposition activity. The released compounds were determined as the total concentrations (μM) of MHET and TPA produced at different incubation times. PET removed from the reaction solution was consecutively washed with distilled water and subsequently vacuum dried for 20 h at 40 °C for scanning electron microscopy (SEM) observation.

**High‐performance liquid chromatography (HPLC) analysis**: The HPLC experiments were performed on an Agilent 1260 Infinity II system equipped with a poroshell EC−C18 column at 35 °C for the analysis of aromatic products. The mobile phase contains water(A)/methanol(B). The separation was carried out using a gradient program of:

(A)=99%and(B)=1%attimet=0

(A)=75%and(B)=25%attimet=15

(A)=0%and(B)=100%attimet=25min

(A)=1%and(B)=99%attimet=26.00min

The flow rate was held constant at 1 mL min^−1^ resulting in a run time of 35 minutes. The aromatic products were detected by the absorbance at 240 nm and recognized according to the retention time of standard compounds. The concentration of each product was calculated in accordance with the calibration curve which was organized from the absorption peak area versus the standard solution concentration. The R‐squared values of the calibration curves were at least 0.996.

**Scanning electron microscopy**: SEM measurements were carried out in a JEOL 7001F equipment operating at 15 kV.

**Crystallinity analyses by differential scanning calorimetry (DSC)**: The crystallinity of untreated and NaOH pretreated PET was measured by DSC (METTLER TOLEDO DSC2). Scans were equilibrated at 0 °C for 1 min, heated to 300 °C at a heating rate of 10 °C⋅min^−1^ and maintained at 300 °C for 1 min, then the sample was cooled to 0 °C at 10 °C⋅min^−1^. All the measurements were carried out in nitrogen atmosphere. The crystallinity was calculated using the equation:

$$X{}_{c}\text{-}DSC\;\left(\%\right)=\;\left[\Delta H{}_{m}-\Delta H{}_{C}/\Delta H\cdot m{}^{0}\right]\times 100$$

where ΔH_m_ is the value of melting enthalpy of the sample (J ⋅ g^−1^), ΔH_c_ is the cold crystallization enthalpy of the sample (J ⋅ g^−1^ ) and ΔHm^0^ is the melting enthalpy of the 100 % crystalline PET (140.1 J ⋅ g^−1^).

## Conflict of interest

The authors declare no conflict of interest.

1

## Supporting information

As a service to our authors and readers, this journal provides supporting information supplied by the authors. Such materials are peer reviewed and may be re‐organized for online delivery, but are not copy‐edited or typeset. Technical support issues arising from supporting information (other than missing files) should be addressed to the authors.

Supporting Information

## Data Availability

The data that support the findings of this study are available from the corresponding author upon reasonable request.
