# Supplementary material for: Accelerated Polyethylene Terephthalate (PET) Enzymatic Degradation by Room Temperature Alkali Pre‐treatment for Reduced Polymer Crystallinity
Source: Chembiochem. 2022 Nov 30;24(1):e202200503. doi: 10.1002/cbic.202200503 (PMC10286761; doi:10.1002/cbic.202200503)
Supplement: Supplementary file 1 — Supporting Information [file CBIC-24-0-s001.pdf]

ChemBioChem

Supporting Information

Accelerated Polyethylene Terephthalate (PET) Enzymatic Degradation by Room Temperature Alkali Pre-treatment for Reduced Polymer Crystallinity

Sariah Giraldo-Narcizo, Nihal Guenani, Ana María Sánchez-Pérez,* and Antonio Guerrero*

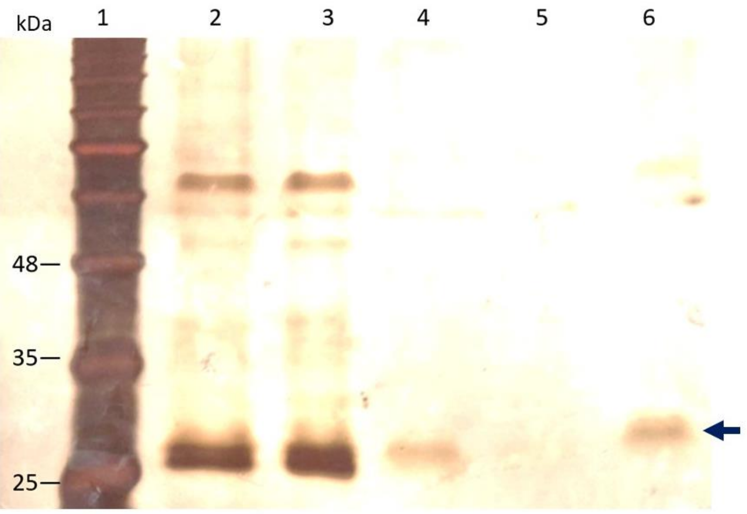

Figure S1. SDS-PAGE analysis for the purification of PETase. Lane 1, Protein Ladder; Lane 2, Elution 1 (Purification His Tag); Lane 3, Elution 2(Purification His Tag); Lane 4, Elution 3 (Purification His Tag); Lane 5, Elution 4 (Purification His Tag); Lane 6, Elution (Purification Size Exclusion).

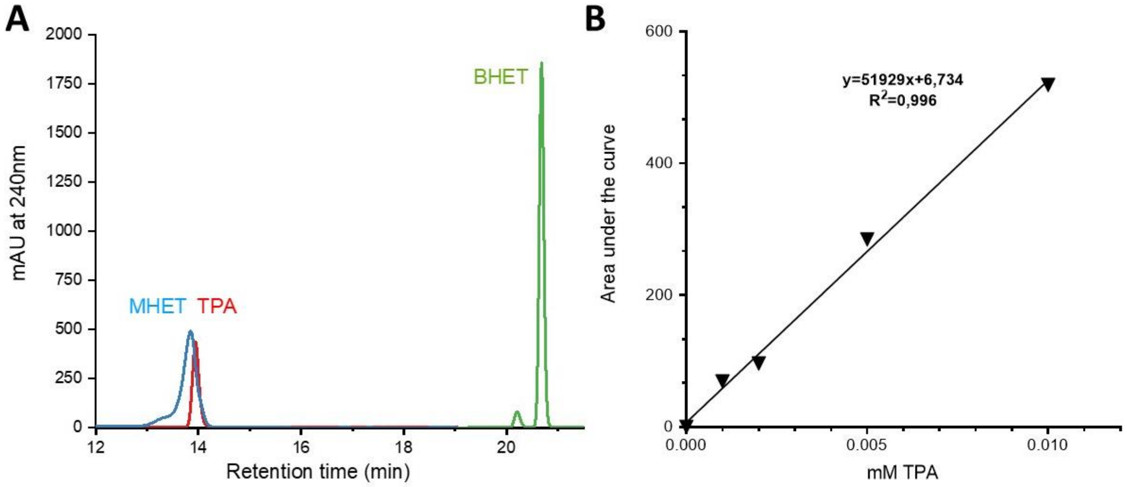

Figure S2. (A) The retention time of monomer products from PETase hydrolysis of BHET (MHET and BHET) and standard solution of TPA detected by HPLC at 240 nm. (B)Standard curves of TPA.
